# Reactivity of Wet scCO_2_ toward Reservoir and Caprock Formations under Elevated Pressure and Temperature Conditions: Implications for CCS and CO_2_-Based Geothermal Energy Extraction

**DOI:** 10.1021/acs.energyfuels.4c04515

**Published:** 2025-01-08

**Authors:** Nicolás Rangel-Jurado, Xiang-Zhao Kong, Anna Kottsova, Luiz Grafulha Morales, Ning Ma, Federico Games, Maren Brehme, Stefano M. Bernasconi, Martin O. Saar

**Affiliations:** ‡ Geothermal Energy and Geofluids Group, Institute of Geophysics, Department of Earth and Planetary Sciences, ETH Zurich, Zurich 8092, Switzerland; ¶ Scientific Centre for Optical and Electron Microscopy (ScopeM), ETH Zurich, Zurich 8093, Switzerland; § Institute of Geochemistry and Petrology, Department of Earth and Planetary Sciences, ETH Zurich, Zurich 8092, Switzerland; ∥ Ad Terra Consultancy, Geneva, 1208, Switzerland; ⊥ Climate Geology, Geological Institute, Department of Earth and Planetary Sciences, ETH Zurich, Zurich 8092, Switzerland; # Computational Geoscience, Geothermics, and Reservoir Geophysics, RWTH Aachen, Aachen 52074, Germany

## Abstract

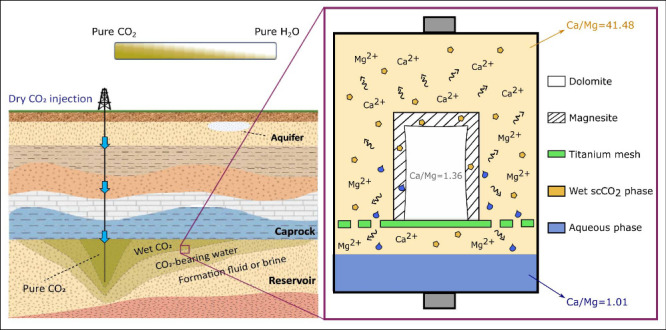

Carbon capture and storage (CCS) and CO_2_-based geothermal energy are promising technologies for reducing CO_2_ emissions and mitigating climate change. Safe implementation of these technologies requires an understanding of how CO_2_ interacts with fluids and rocks at depth, particularly under elevated pressure and temperature. While CO_2_-bearing aqueous solutions in geological reservoirs have been extensively studied, the chemical behavior of water-bearing supercritical CO_2_ remains largely overlooked by academics and practitioners alike. We address this knowledge gap by conducting core-scale laboratory experiments, focusing on the chemical reactivity of water-bearing supercritical CO_2_ (wet scCO_2_) with reservoir and caprock lithologies and simulating deep reservoir conditions (35 MPa, 150 °C). Employing a suite of high-resolution analytical techniques, we characterize the evolution of morphological and compositional properties, shedding light on the ion transport and mineral dissolution processes, caused by both the aqueous and nonaqueous phases. Our results show that fluid–mineral interactions involving wet scCO_2_ are significantly less severe than those caused by equivalent CO_2_-bearing aqueous solutions. Nonetheless, our experiments reveal that wet scCO_2_ can induce mineral dissolution reactions upon contact with dolomite. This dissolution appears limited, incongruent, and self-sealing, characterized by preferential leaching of calcium over magnesium ions, leading to supersaturation of the scCO_2_ phase and reprecipitation of secondary carbonates. The markedly differing quantities of Ca^2+^ and Mg^2+^ ions transported by wet scCO_2_ streams provide clear evidence of the nonstoichiometric dissolution of dolomite. More importantly, this finding represents the first reported observation of ion transport processes driven by water continuously dissolved in the scCO_2_ phase, which challenges prevailing views on the chemical reactivity of this fluid and highlights the need for further investigation. A comprehensive understanding of the chemical behavior of CO_2_-rich supercritical fluids is critical for ensuring the feasibility and security of deep geological CO_2_ storage and CO_2_-based geothermal energy.

## Introduction

Over the past two centuries, global population growth, along with rising energy consumption per capita, has driven a dramatic increase in primary energy demand, predominantly met by fossil fuels, leading to a substantial rise in atmospheric carbon dioxide (CO_2_) concentrations. The global reliance on fossil fuels to sustain development and our inability to deal with the resulting greenhouse gas emissions have caused serious concerns about climate change and its largely irreversible impact on the environment.^1^ As a result, significant efforts are being directed toward carbon reduction and renewable energy technologies aimed at substantially reducing CO_2_ emissions into the atmosphere. Various CO_2_ sequestration strategies have been identified across the terrestrial,^2−4^ oceanic,^5−7^ and geological domains.^8,9^ Among the geological options, two prominent examples are carbon capture and storage (CCS)^10^ and CO_2_-based geothermal energy extraction; the latter includes subtypes such as CO_2_-based Enhanced Geothermal Systems (CO_2_-EGS)^11^ and CO_2_-Plume Geothermal (CPG) systems.^12^

CCS is a promising technology that involves offsetting anthropogenic CO_2_ emissions by capturing the emitted carbon dioxide from large point sources, such as cement manufacturers, biofuel refineries, and fossil-fueled power plants. The captured CO_2_ is then transported and permanently stored in a high-density state within deep geological formations underground, such as deep saline formations or (partially) depleted hydrocarbon fields, preventing its release to the atmosphere.^13,14^ In recent years, CCS development has intensified, predominantly in North America and Western Europe, in an effort to reach 5–10 gigatons of CO_2_ storage capacity per year by 2050.^13,15,16^ However, despite its technological maturity, sociopolitical and economic constraints have limited the CCS storage capacity to only 50 megatons worldwide so far^17^—2 orders of magnitude below the goal for 2050. Two major obstacles currently limiting CCS deployment are public acceptance and the absence of a clear, long-standing, and reliable revenue model to attract investment.^18−20^

Rather than considering the CO_2_ as a disposal fluid exclusively, CO_2_-based geothermal energy systems offer a complement to CCS by using injected CO_2_ to extract geothermal energy while simultaneously allowing for its permanent geological storage. In the supercritical state, CO_2_ displays unique transport properties, including gas-like viscosity and liquid-like density, i.e., low kinematic viscosity, which can significantly increase the heat extraction rate from geothermal reservoirs^11,12,21,22^ and increase the heat-to-electricity conversion efficiency via the introduction of a direct-CO_2_ turbine.^23,24^ Moreover, harnessing heat from geological reservoirs designated for CO_2_ storage increases the density of injected CO_2_. This heightened fluid density can substantially enhance the CO_2_ storage potential of a CPG system compared to an analogous CCS scenario without geothermal energy extraction.^25,26^

When reservoir conditions reach ≥7.38 MPa and ≥31.10 °C, the injected CO_2_ is at its supercritical state (hereafter scCO_2_) and forms buoyant plumes with enhanced transport^11,21,27,28^ and solvent^29,30^ properties. These buoyant scCO_2_ plumes are expected to displace *in situ* formation fluids, preferentially flowing through the upper section of the reservoir due to their buoyancy, where the scCO_2_ comes in direct contact with the caprock.^31,32^ Compared to water-based geothermal energy extraction, the chemical inertness of pure or *dry* CO_2_ toward mineral surfaces was initially identified as a distinct operational advantage.^11,27^ A geothermal fluid that is chemically inert would minimize the potential for dissolution reactions in the subsurface, thereby reducing the risk of mineral scaling in wellbores and surface facilities.

**Figure 1:**
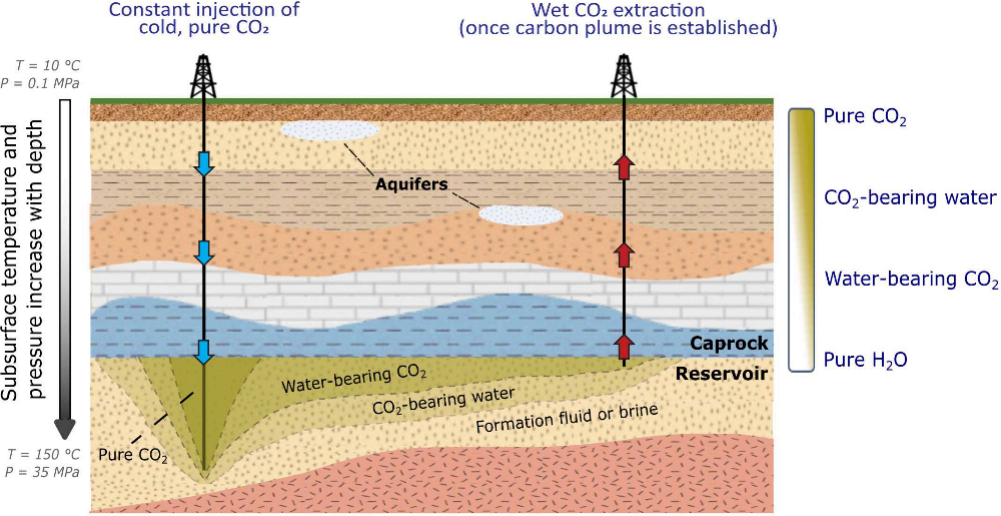
Schematic diagram showing supercritical carbon dioxide (scCO_2_) injection in a deep water/brine-filled formation. Subsurface temperature and pressure increase with depth, typically exceeding 100 °C and 25 MPa beyond 3 km. The values shown below the black arrow represent the experimental conditions used in this study, simulating a reservoir depth between 3.5 and 4 km. CO_2_ injection creates regions that exhibit a full spectrum of mutual scCO_2_–H_2_O solubility. Pure scCO_2_ and water-bearing (or wet) scCO_2_ are less dense than the aqueous phases for nearly all pressure and temperature conditions in subsurface formations, resulting in the upward migration of the scCO_2_-rich phase toward the caprock.

However, while it is commonly recognized that pure CO_2_ is generally unreactive toward rocks comprised of silicate and carbonate minerals,^32−34^ it is likely inaccurate to assume that the injected CO_2_ will remain *dry* and chemically inert once introduced into geological reservoirs.^29,35,36^ Upon injection, mutual dissolution occurs between the CO_2_ phase and formation fluids (often water or brine) across their interface: CO_2_ dissolves into the formation water, while some of the water vaporizes into the scCO_2_ phase.^37,38^ This mixing interaction creates regions that span across the full CO_2_–H_2_O mutual solubility spectrum, which can be considerably more reactive than anhydrous CO_2_ and can trigger a broad range of reactions.^29,32,33^ The specific fluid compositions along this mutual solubility spectrum are pressure- and temperature-dependent, varying laterally and vertically with the distance from the high-pressure, low-temperature CO_2_ injection source (Figure 1). As a result, dry CO_2_ is likely confined to the near-wellbore injection region, while the water content tends to increase in the far-field. Accordingly, chemical interactions between various CO_2_–H_2_O mixtures and rocks are expected to occur nearly throughout the entire reservoir.

Empirical research on geological CO_2_ storage indicates that injecting scCO_2_ into saline formations disrupts the chemical equilibrium between the brine and the mineral assemblages.^39−42^ The extent and nature of these geochemical interactions exhibit a significant degree of variability because the phase saturations and chemical compositions differ at various locations relative to the scCO_2_ injection well.^35,43^ Notably, the vast majority of literature on this matter considers aqueous reactions exclusively.^39,44−52^ In the aqueous phase (i.e., fluid mixtures where water is the solvent), the dissolution of CO_2_ forms carbonic acid, which then dissociates to form hydrogen and bicarbonate ions (eq 1a) that have the potential to induce mineral dissolution reactions. In the example below, calcite is in equilibrium with carbonic acid, as well as calcium and bicarbonate ions (eq 1b).
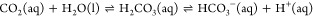
1a
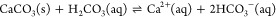
1b

As a result of these reactions, CO_2_-bearing water or brine displays a heightened reactivity toward mineral surfaces, attributable to the reduction in pH, which induces the dissolution of primary rock-forming minerals and, in some cases, the subsequent precipitation of secondary minerals. Several studies have reported on the role that CO_2_-bearing aqueous solutions play in the dissolution/reprecipitation of carbonate and silicate minerals,^53−55^ feldspars,^56,57^ and clay minerals.^58,59^ Additionally, experiments conducted on carbonate-cemented rocks also show a drastic deterioration in mechanical strength, induced by CO_2_-enriched brines.^60,61^

**Figure 2:**
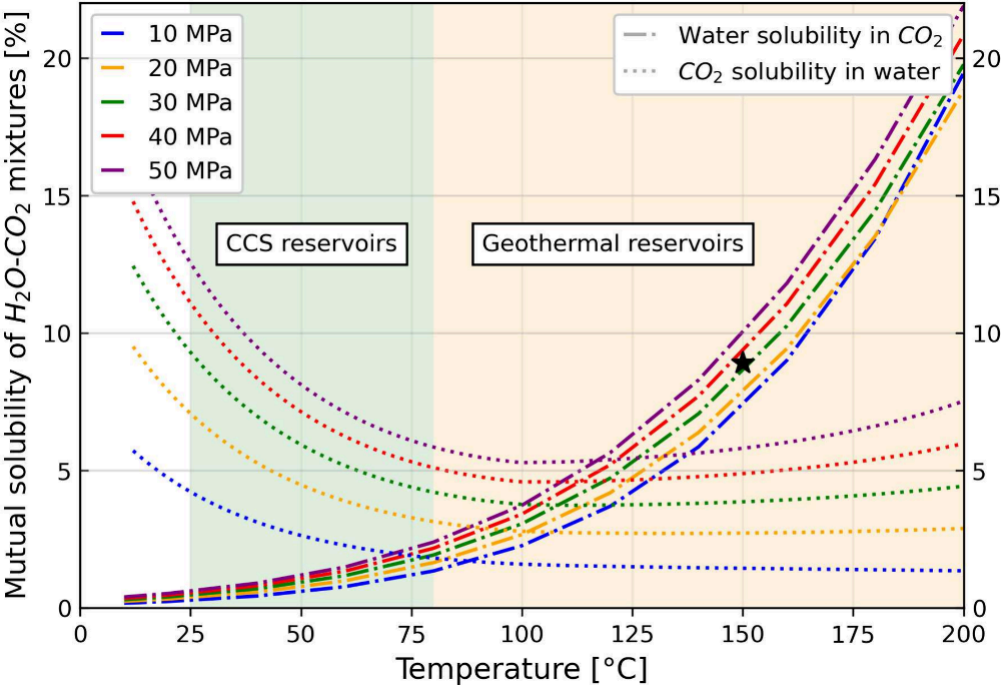
Mutual solubility of H_2_O–CO_2_ mixtures in mol % as a function of pressure and temperature. The solubility data depicted in this figure were derived using the model by Aavatsmark and Kauffman^68^ for water solubility in CO_2_ and the model by Spycher and Pruess^29^ for CO_2_ solubility in water. The black star indicates the scope of our experimental study, which focuses on water-saturated CO_2_ and reflects the thermodynamic conditions of a mid- to high-enthalpy geothermal reservoir (150 °C and 35 MPa). Note that CPG reservoirs are geothermal reservoirs as well as a (higher-temperature and higher-pressure) subset of CCS reservoirs.

Despite this extensive coverage of H_2_O–CO_2_ mixtures in the earth science literature, important limitations remain. First, chemical processes occurring within the nonaqueous phase (i.e., solutions in which CO_2_ serves as the solvent) have historically been overlooked in both CCS- and CPG-related research. Second, studies have predominantly focused on the influence of relatively low-pressure and temperature conditions, relevant to shallow CCS projects (<2 km). However, considering elevated pressure and temperature conditions (>30 MPa, >100 °C) is essential when somewhat deeper (2–5 km) reservoirs are to be employed for CCS and a necessity to enable the addition of CPG.^23,62^ Interestingly, while the solubility of CO_2_ in water tends to decrease with an increase in temperature, particularly for midenthalpy reservoirs up to 150 °C, the solubility of water in CO_2_ increases exponentially with temperature (Figure 2). Therefore, the significance of water-bearing scCO_2_ might prevail in deep subsurface applications. Nonetheless, to the best of our knowledge, most, if not all, numerical models developed to date for simulating geochemical interactions in CCS, CO_2_-EGS, and CPG systems have exclusively focused on aqueous reactions,^63−67^ leaving the potential impact of mineral dissolution induced by nonaqueous phases computationally unexplored.

Although limited and more recent, empirical research in the field of nonaqueous CO_2_–H_2_O mixtures has found that mineral exposure to water-bearing scCO_2_ (here referred to as *wet* scCO_2_) can trigger reactions of equal importance to processes occurring in aqueous-dominated systems.^33,69^ The prevailing hypothesis that these investigations pose is that the dissolved water in the scCO_2_ phase partially exsolves and creates a thin liquid film (at the μm- to nm-scale) that adsorbs onto the mineral surfaces.^70−77^ This exsolved water film is hypothesized to allow for the formation of carbonic acid and accommodate a series of aqueous reactions that induce chemical and mechanical transformations in the host rock and wellbore materials in the vicinity of the reacted surface.^75,78^ Reported alterations of physical–chemical properties include the increased reactivity of wet scCO_2_ toward surrounding steel and other wellbore materials,^79^ slight variations in matrix porosity and permeability along reaction surfaces,^80^ mineral replacement in silicate and carbonate rocks,^36,71,72^ hydration and carbonation of existing minerals,^81^ CO_2_ diffusion into and out of clay-rich caprocks, resulting in smectite swelling/shrinkage,^71,72,82^ mild geomechanical weakening of sandstones,^41^ and dissolution, followed by precipitation, of amorphous minerals.^31,32,83^ Note that the vast majority of these observations were reproduced under the thermodynamic conditions typically considered for CCS projects (≤25 MPa, ≤ 100 °C); thus, the extent of chemical reactions when considering a broader range of reservoir conditions (e.g., geothermal reservoirs; see also Figure 2) might have been underestimated.

In this study, we investigate the geochemical behavior of scCO_2_–H_2_O mixtures toward different lithologies, with a particular focus on the reactivity of wet scCO_2_ under elevated pressure and temperature conditions. Core-scale batch reaction experiments were performed on two rock specimens from the Muschelkalk and Gipskeuper formations of northeastern Switzerland over several days. These lithologies are of significant national interest to Switzerland, as they have been identified as potential targets for CCS^84^ and geothermal energy extraction,^85,86^ among other subsurface applications. A suite of high-resolution analytical techniques was used to characterize the evolution of petrophysical, morphological, and compositional properties of the rock specimens following exposure to wet scCO_2_. Broadening the understanding of the geochemical reactivity of scCO_2_–H_2_O mixtures across a wide range of pressure and temperature conditions is critical for ensuring the long-term safety of CCS and demonstrating the technical viability of the CO_2_-based geothermal energy extraction.

## Materials and Methods

### Sample Description

Experiments were conducted on core-scale specimens from the Muschelkalk and Gipskeuper formations from Northeastern Switzerland. The Muschelkalk formation, a middle unit of the Triassic period, dating back to approximately 247 to 235 million years, is predominantly characterized by marine limestones and dolomites. The Gipskeuper, or Upper Keuper, is a younger formation from the late Triassic, roughly 235 to 201 million years old, consisting largely of gypsum- and anhydrite-bearing clays and carbonates, reflecting more terrestrial and arid conditions. The formations of interest dip to the Southwest of the country, reaching depths of nearly 4 km in specific locations of the Swiss Molasse Basin and, due to their favorable petrophysical properties and stratigraphic correlation, have been identified as targets for CCS and/or geothermal energy extraction—using water-based or CO_2_-based energy-extraction technologies.^84−87^ The Muschelkalk rock specimen (Sample 822.72A) was obtained from the Weiach wellbore,^88^ whereas the Gipskeuper specimen (Sample 728.18) was obtained from the Benken wellbore.^89^ Both wellbores were drilled in the Zurich canton by *NAGRA*.

The Muschelkalk formation, generally more permeable and porous and found at greater depths than the Gipskeuper formation,^84^ has been identified as a potential reservoir for various subsurface applications, ranging from radioactive waste disposal and CO_2_ storage to geothermal energy extraction. The Gipskeuper, a rock formation that overlies the Muschelkalk across the Molasse basin, has been identified as a regional caprock. Hence, throughout this text, Sample 822.72A, corresponding to the Muschelkalk formation, is referred to as the reservoir rock, whereas Sample 728.18, from the Gipskeuper formation, is referred to as the caprock.

Both rock specimens, cylindrical in shape and similar in size (2.53 ± 0.01 cm in diameter and 2.86 ± 0.03 cm in length), are relatively homogeneous and compact, with no observable fractures. A wide range of high-resolution techniques were employed before and after the fluid–mineral interactions occurred to characterize the evolution of the morphology, petrophysical properties, and chemical composition of the samples. Descriptions of the specific analyses employed are provided in the following subsections below.

### Batch Reaction: Exposure to Wet scCO_2_

**Figure 3:**
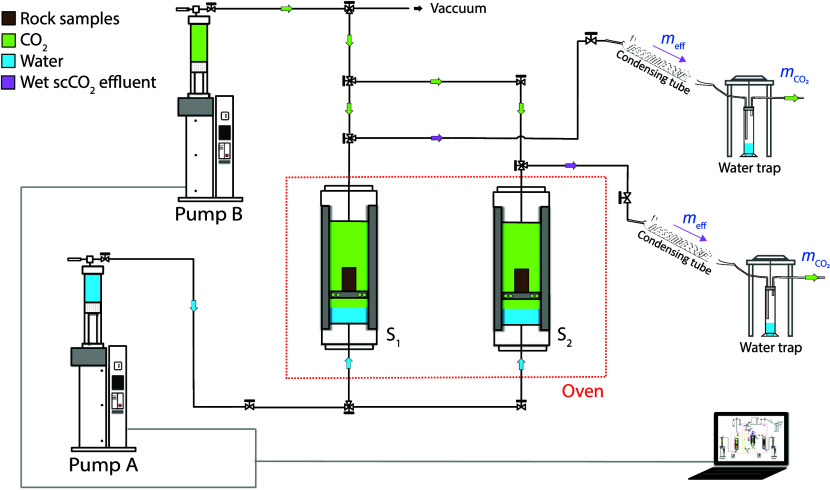
Schematic of the experiment setup. Batch reactors S1 and S2 contain the reservoir and the cap rock, respectively. Rock specimens are surrounded by the scCO_2_ phase and separated from the aqueous solution by a titanium holder.

As indicated in the Introduction, in this study, scCO_2_ is intended to be the primary solvent of dissolved water. Such a consideration allows for the investigation of the buoyant, water-bearing scCO_2_ plume, which flows through the top part of the reservoir formation, where it may come into contact with the caprock. To achieve this, the following experimental procedures were conducted. After being dried for 24 h in an oven at 60 °C, each rock specimen was placed in a cylindrical titanium holder with a solid base plate and housed in a 500 mL batch reactor, composed of stainless steel, as shown in Figure 3. Several 5 mm holes were drilled through the margin of the base plate (region between the cylinder and reactor walls) to allow fluid exchange across the base. Before placing the specimens in the batch reactors, both batch reactors and the titanium holder underwent a thorough triple-rinsing process with hydrochloric acid and Milli-Q water. A total of 30 mL of Milli-Q water was added to the bottom of each batch reactor, filling a volume, located a few centimeters below the titanium holder, such that the rock specimens remained separated from the liquid water during the experiments. Subsequently, the batch reactors were vacuumed to remove air in the remaining volume and specimens and the dissolved air in the Milli-Q water. Next, liquid CO_2_ with a >99.99% purity was introduced into the reactors from the reactor top using a high-performance volumetric pump (Model Stigma 300D). Finally, the batch reactors were placed inside an oven for temperature control.

The pressure and temperature of the batch reactors were simultaneously increased beyond the critical point of CO_2_, until they reached values of 35 MPa and 150 °C, respectively. Here, the pressure was controlled by adding or removing CO_2_ from the reactors. The simultaneous increase of pressure and temperature causes the dissolution of CO_2_ into the water phase and the dissolution of water into the scCO_2_ phase through evaporation, turning dry scCO_2_ into wet scCO_2_, which can later react with the rock specimens. Under our experimental temperature and pressure conditions, the expected mole fraction solubility of CO_2_ in water is 0.044, as calculated using the solubility model of Spycher and Pruess,^29^ while the solubility of water in CO_2_ is 0.089, based on the model of Aavatsmark and Kaufmann.^68^ The latter solubility value implies that only ∼9.8 mL of water will vaporize into the scCO_2_ phase, leaving ∼20.2 mL of liquid water at the reactor bottom and ∼420 mL of wet scCO_2_ in the remaining volume of the 500 mL reactor. The excess water at the reactor bottom ensured the saturation of water in scCO_2_ throughout the entire duration of the experiment.

**Figure 4:**
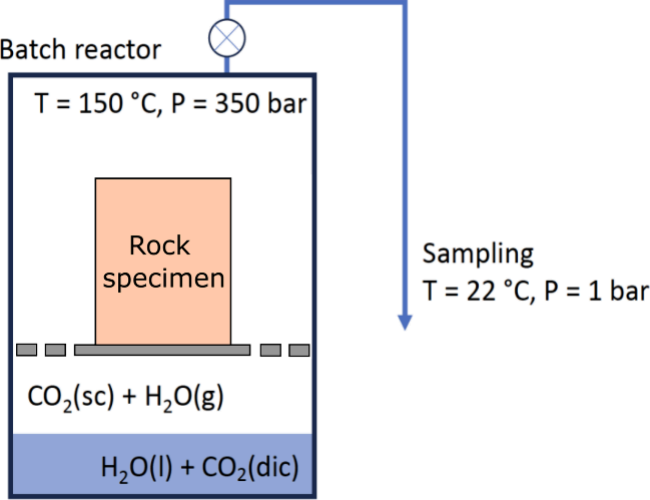
Schematic batch reaction of wet CO_2_ with the rock specimens. Here, CO_2_(sc) refers to supercritical CO_2_, H_2_O(l) refers to liquid water, H_2_O(g) refers to dissolved water in CO_2_(sc), and CO_2_(dic) refers to total dissolved inorganic carbon (dic = CO_2_(aq) + HCO_3_^–^(aq) + CO_3_^2–^(aq)) in water. A titanium mesh was used to separate the rock specimen from the CO_2_-saturated water at the bottom of the batch reactor.

During the experiment, the batch reactors were continuously monitored and recorded for pressure, temperature, and volume, using Falcon, a Sanchez control software. Throughout the 500 h experiment, the pressure remained within 35 ± 1 MPa, while the temperature consistently maintained at 150 ± 3 °C. Notably, no significant fluid leaks were observed. A sampling apparatus was installed at the upper outlets of the batch reactors to collect the wet CO_2_ effluents immediately before terminating the experiment. This apparatus employed a high-pressure condensing tube and water traps, facilitating the separation of condensed water from the gaseous CO_2_ phase and its subsequent release (Figures 3 and 4).

### Stable Isotope Analysis

The stable ^18^O isotope was employed as a tracer to monitor interactions among liquid water, water dissolved in scCO_2_, the CO_2_ phase, and rock specimens. By artificially enriching the ^18^O content of the Milli-Q water, the objective was to assess whether the water located at the bottom of the batch reactors vaporized into the scCO_2_ phase and subsequently engaged in interactions with the rock specimens. To accomplish this, ^18^O of the deionized (Milli-Q) water was enriched from its original δ^18^O = −11 to approximately δ^18^O = +100 on the VSMOW (Vienna Standard Mean Ocean Water) scale. In comparison, the δ^18^O values of our CO_2_ and the rock specimens were measured to be +15 and +28 ± 1, respectively. The carbon isotopes were not enriched or otherwise modified at the start of our experiment.

Samples of rock powders (from specimen surfaces), liquid aliquots, and gas aliquots were collected both before and after exposure to wet CO_2_ and analyzed at the Geological Institute in the Department of Earth and Planetary Sciences, ETH Zurich. These analyses were conducted using a ThermoFinnigan *GasBench II* equipped with an autosampler and a Thermo Fischer Scientific Delta V Plus mass spectrometer. The isotope composition of rock carbonate powders was obtained following the experimental procedure described in ref (90). To determine the isotope composition for CO_2_, 50–70 μL of gaseous CO_2_ was injected into 12 mL septum-capped borosilicate vials previously flushed with high-purity helium. The *GasBench II* and Delta V Plus mass spectrometer were calibrated by measuring three laboratory-internal CO_2_ gases calibrated to the international reference materials RM8562, RM8563, and RM8564 distributed by NIST. The results are reported in the conventional delta notation with respect to VSMOW. The isotope composition of aqueous samples was measured following the CO_2_ equilibration method. During the measurements, 200 μL of water is pipetted into 12 mL septum-capped vials which are subsequently filled with a mixture of 0.3% (vol.) CO_2_ and He. After reaching equilibration at 25 °C for at least 18 h, the CO_2_–He mixture is measured using the gas bench connected to the mass spectrometer. The system was calibrated employing the international standards VSMOW (0‰), SLAP (−55.5‰), and GISP (−24.78‰). The results are reported in the conventional delta notation with respect to VSMOW. Reproducibility of the measurements, based on repeated measurements of an internal standard, was better than 0.1 ‰.

We consider equilibrium isotopic partitioning conditions in the batch reactor at a temperature of *T* = 150 ±3 °C and a pressure of *p* = 35 ± 1 MPa.

### X-ray Diffraction

Powder X-ray diffraction (XRD) measurements were performed to determine which crystallized mineral phases had originally been present in the rock specimens and which ones were potentially created when conducting batch experiments. To achieve this, approximately 50 mg of rock powder, located close to the exposed reaction surface of each rock specimen, was collected and pulverized, producing a particle size of ∼10 μm. The pulverized samples were then homogenized and loaded on a low-background single-crystal silicon holder. XRD measurements were conducted using an Empyrean 3 diffractometer (Malvern Panalytical) equipped with a Cu source (Cu Kα = 1.5406 Å, 40 kV, 40 mA) and secondary graphite monochromator at the Institute of Geochemistry and Petrology, Department of Earth and Planetary Sciences, ETH Zurich. The diffraction patterns were continuously collected from 5 to 90° (2θ) with a step size of 0.013° and a 65 s dwell time at ambient temperature using a PIXcel1D detector. Phase identification and Rietveld refinements were carried out, using HighScore software and the Crystallography Open Database (COD), to investigate the mineralogy and estimate mineral modal abundances.

### Scanning Electron Microscopy

Scanning electron microscopy (SEM) imaging was employed to image the surfaces of the rock specimens before and after the experiments. This technique enables the determination of the general mineralogy and the examination of microstructural features and textures located on the reaction surfaces. Additionally, SEM when coupled with other analytical techniques such as energy-dispersive spectroscopy (resulting in SEM-EDS) can determine the chemical composition of mineral phases and provide insights into the mobility (enrichment and depletion) of rock-forming elements. SEM images were acquired in a Hitachi SU5000 FEG SEM instrument at the Scientific Centre for Optical and Electron Microscopy at ETH Zurich. Secondary and backscattered electron images were obtained for uncoated surfaces under low-vacuum conditions, with nitrogen-controlled pressures varying between 50 and 100 Pa, at 10 kV and a beam current of 1.3 nA. SEM-EDS mapping was also performed, with the same imaging conditions for element identification, using an Oxford EDS detector, controlled by Aztec software (v. 5.1).

Since dolomite is abundant and expected to be the most reactive mineral phase in both the Muschelkalk and Gipskeuper samples, the molar ratio of calcium and magnesium (i.e., Ca/Mg stoichiometric ratio) is calculated to assess the compatibility of these two elements across different phases.

### Inductively Coupled Plasma Atomic Emission Spectroscopy

Upon termination of the batch experiments, fluid effluents of both the aqueous and gaseous/supercritical phases were analyzed by inductively coupled plasma atomic emission spectroscopy (ICP-AES). This analysis was conducted to determine the extent of the associated ion dissociation and transport processes, induced by the wet scCO_2_ and the exsolved aqueous solution. For this, five fluid samples were sent to *FILAB* for analysis. There, samples were diluted to 1/10th and 1/100th of their original volume by acidifying them with 10% HNO_3_ (final concentration of HNO_3_ in each sample: 1% HNO_3_) and analyzed using FILAB’s internal methodology.

## Results and Discussion

Upon completion of the 500 h batch experiments at 35 MPa and 150 °C, analytical techniques were once again employed to reveal the petrophysical, geophysical, geochemical, and microstructural alterations, incurred by each rock sample as a consequence of their interaction with wet scCO_2_. The ensuing sections provide a synthesis of our experiment observations and an interpretation of the thermal, hydraulic, and chemical processes associated with these observations.

### Baseline Information

The petrophysical properties of two rock specimens, labeled 728.18 and 822.72A, were analyzed prior to interaction with wet scCO_2_ under elevated pressure and temperature conditions. Specimen 728.18, corresponding to the Gipskeuper lithological unit (caprock), exhibited a density of 2.95 g/cm^3^, a mass of 40.18 g, a porosity of 0.6%, and a permeability of 2.3 × 10^–20^ m^2^. Specimen 822.72A, associated with the Muschelkalk lithological unit (reservoir rock), displayed a density of 2.18 g/cm^3^, a mass of 29.41 g, a porosity of 22%, and a permeability of 1.64 × 10^–15^ m^2^.

XRD and SEM analyses reveal that, on average, the chemical composition of the bulk unreacted caprock (Gipskeuper specimen) originally consists of dolomite (54.4%), anhydrite (37.8%), and minor quantities of celestine (5.1%), quartz (2.7%), and various trace elements. In contrast, the unreacted reservoir rock (Muschelkalk sample) is composed of pure dolomite (>99.9%) with the occasional presence of trace elements, such as sodium (Na) and potassium (K). The elemental compositions of both samples, in terms of weight percentage and mole fraction, are presented in Tables 1 and 2.

**Table 1:** Elemental Composition of the Unreacted Muschelkalk (Reservoir) Sample, as Determined by SEM Scans

	O	Ca	Mg	Na	K	Si	S	Fe	total
Molar mass (g/mol)	16.00	40.08	24.30	22.98	39.00	28.00	32.10	56.00	
Avg. weight (%)	54.21	31.05	13.84	0.30	0.36	0.12	0.12	0.00	100.0
Mole fraction (−)	0.71	0.16	0.12	0.00	0.00	0.00	0.00	0.00	1.00

**Table 2:** Elemental Composition of the Unreacted Gipskeuper (Caprock) Sample, as Determined by SEM Scans

	O	Ca	Mg	Na	K	Si	S	Fe	Al	W	Sr	total
Molar mass (g/mol)	16.00	40.08	24.30	22.98	39.00	28.00	32.10	56.00	26.9	183.84	87.6	
Avg. weight (%)	43.53	28.93	5.22	0.13	0.00	3.29	16.65	0.30	0.27	0.56	0.33	99.21
Mole fraction (−)	0.63	0.17	0.05	0.00	0.00	0.03	0.12	0.00	0.00	0.00	0.00	1.00

**Figure 5:**
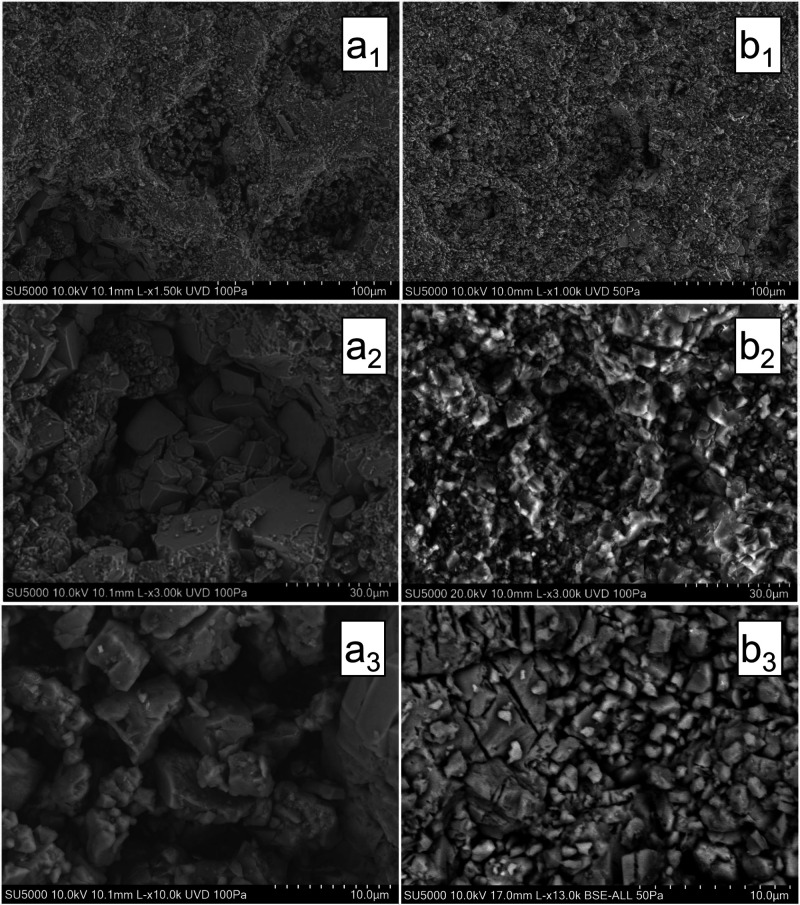
Scanning electron microscopy (SEM) images of the Muschelkalk sample. With the exception of image b_3_ (backscatter image), all other images are secondary electron images. Images labeled with “a” portray the unreacted specimen, whereas images labeled with “b” depict the exposed surface of the specimen after the batch reactions with the wet scCO_2_ occurred. Each row uses a similar field of view with magnification increasing from a_1_,b_1_ to a_3_,b_3_ to focus on the pore structures.

**Figure 6:**
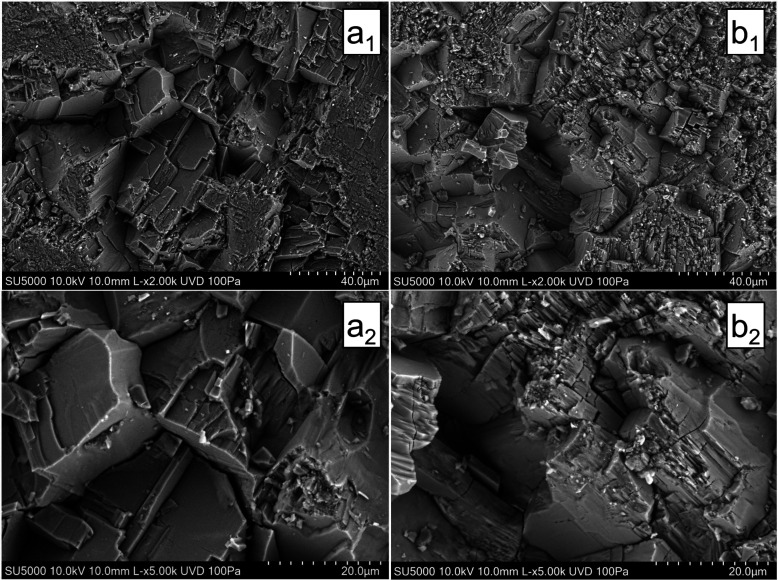
Scanning electron microscopy (SEM) images of the Gipskeuper sample. Images labeled with an “a” portray the unreacted specimen, whereas images labeled with “b” depict the exposed surface of the specimen after the batch reactions with the wet scCO_2_ occurred. Each row uses a similar field of view with magnification increasing from a_1_,b_1_ to a_2_,b_2_.

The left-hand panels (a_1_–a_3_) in Figure 5 exhibit the original morphological characteristics of the Muschelkalk sample, revealing a dolomitic matrix with a highly porous structure. The pores are well-defined and vary in size, with some reaching up to 50 μm in diameter. The overall texture is granular, and the matrix is predominantly composed of euhedral dolomite crystals with distinct geometric boundaries, suggesting a high degree of mineralization. In stark contrast, the Gipskeuper sample initially exhibited markedly different structure and texture. Unlike the porous Muschelkalk specimen, the Gipskeuper specimen is characterized by a compact and densely packed matrix devoid of visible pores (Figure 6). The spaces between the angular mineral grains are entirely filled with cementing material, which was identified as anhydrite through SEM-EDS. This tightly interlocked arrangement of dolomite and anhydrite creates a robust barrier to fluid migration, which explains the exceptionally low porosity and permeability measured in this rock specimen. The observed disparities in pore size, structural arrangement, and chemical composition draw attention to distinctive geological features between the reservoir and caprock specimens, which are anticipated to influence their respective geochemical behaviors.

### Petro- and Geophysical Alterations

Despite interacting with wet scCO_2_ under elevated pressure and temperature conditions for several weeks, both the Muschelkalk and Gipskeuper samples exhibit only a subtle loss of mass. The Muschelkalk sample experienced a mass loss of 0.10 g, equivalent to 0.35% of its original mass, while the Gipskeuper sample showed a loss of 0.01 g, representing 0.02% of its original mass. It is important to note that not all of the mass loss can be attributed to mineral dissolution exclusively, as some matrix weakening might have occurred during the experiment and minor fragments may have been scraped off during the removal of samples from the titanium mesh holders.

Both rock specimens exhibit a mild increase in porosity from 22% to 25% for the Mushelkalk sample and from 0.6% to 0.8% for the Gipskeuper specimen. The bulk permeabilities of the samples were not significantly modified during the experiments. The postreaction permeability measurements stay constant, within the experiment uncertainty, at values of 1.6 × 10^–15^ and 2.3 × 10^–20^ m^2^ for the Muschelkalk and the Gispekeuper specimens, respectively.

While geochemical interactions had a quantifiable impact on the petrophysical properties of both rocks, as indicated by the observed changes in mass and porosity, their extent appears to be minor.

### Morphological Alterations

SEM micrographs, portraying the pre- and postexperiment states of the rock specimens, are shown in Figure 5 for the Muschelkalk sample and in Figure 6 for the Gipskeuper sample. The panels on the left-hand side (a_1_–a_3_) of Figure 5 depict unreacted dolomite fragments, which originally display a euhedral habit characterized by sharp, well-formed crystal edges and a distinct granular, yet porous texture. In contrast, the micrographs of the reacted specimens on the right-hand side panels (b_1_–b_3_) illustrate the surface morphology of the reacted specimens after high-pressure and high-temperature reactions occurred. These images show subhedral mineral fragments with reduced grain size and partially rounded edges, suggesting dissolution and recrystallization processes. The once-prominent pore structures observed in the unreacted specimens now appear significantly modified, with the pore spaces partially to fully occupied by newly precipitated material of finer grain size. This new phase, likely resulting from mineral precipitation during or right after the bath reaction concluded.

Examination of pre- and postexperiment SEM micrographs of the Gipskeuper sample (Figure 6) reveals characteristics analogous to those described above, where distinct mineral edges have undergone alteration and rounding, while finer-grained precipitates have formed on the mineral surfaces and at the intersections of pre-existing phases. However, the extent of morphological alteration in the Gipskeuper sample is comparatively lower than that observed in the Muschelkalk reservoir rock. This difference is attributed to the mineralogical composition of the Gipskeuper sample, which contains a higher proportion of noncarbonate phases, such as anhydrite and quartz. These minerals appear to be significantly less reactive under the experimental conditions employed, thereby reducing the overall degree of dissolution and recrystallization; as a result, the crystal habit of the Gipskeuper specimen remains largely intact (Figure 6).

The observed variability in morphological alterations of the reacted mineral surfaces provides compelling evidence of fluid–mineral interactions. These findings emphasize the critical role of mineralogical variability in controlling the extent and nature of wet scCO_2_ reactivity, which manifests itself through its impact on the dissolution, precipitation, and textural modifications of reservoir and caprock lithologies.

### Compositional Alterations

#### Fractionation of Stable Isotopes

In our experiments, we separate fluid samples into three distinct categories: water samples collected from the aqueous solution at the reactor bottom, effluent samples of wet scCO_2_, and solid powder samples obtained from rock specimens. It is worth noting that fluid samples encompass both the sampled wet scCO_2_ phase and the condensed aqueous solution that remained in the reactor. It is anticipated that water exsolution from the wet scCO_2_ phase occurs due to the decrease in the pressure (during effluent sampling and later termination of experiments) and temperature (termination of experiments). We argue that the majority of water condensation occurs during the experiment termination, as the solubility of water in scCO_2_ only experiences a slight decrease due to the pressure reduction from 35 to 5 MPa at 150 °C during effluent sampling (Figure 2). Upon contact with the rock specimens, the condensed water, charged with CO_2_, is expected to induce mineral dissolution, as previously reported in related studies.^33,75,78^ Nonetheless, isotope fractionation is anticipated among the residual water at the reactor bottom, scCO_2_, water dissolved in the scCO_2_ phase, CO_2_ dissolved in water, and solid minerals. Just before the experiments were terminated, fluid effluents of the wet CO_2_ phase were collected using the sampling apparatus described in Materials and Methods. Subsequently, the system’s pressure and temperature were gradually reduced, leading to the exsolution of the water dissolved in the scCO_2_ phase. This condensed water likely came into contact with the rock specimens and then settled at the bottom of the batch reactors, mixing with the residual liquid water. Samples of this aqueous solution, rock powders (sourced from the samples’ exposed surfaces), and the free-phase CO_2_ gas were also collected and subjected to analyses to test the hypothesis of isotope fractionation.

**Figure 7:**
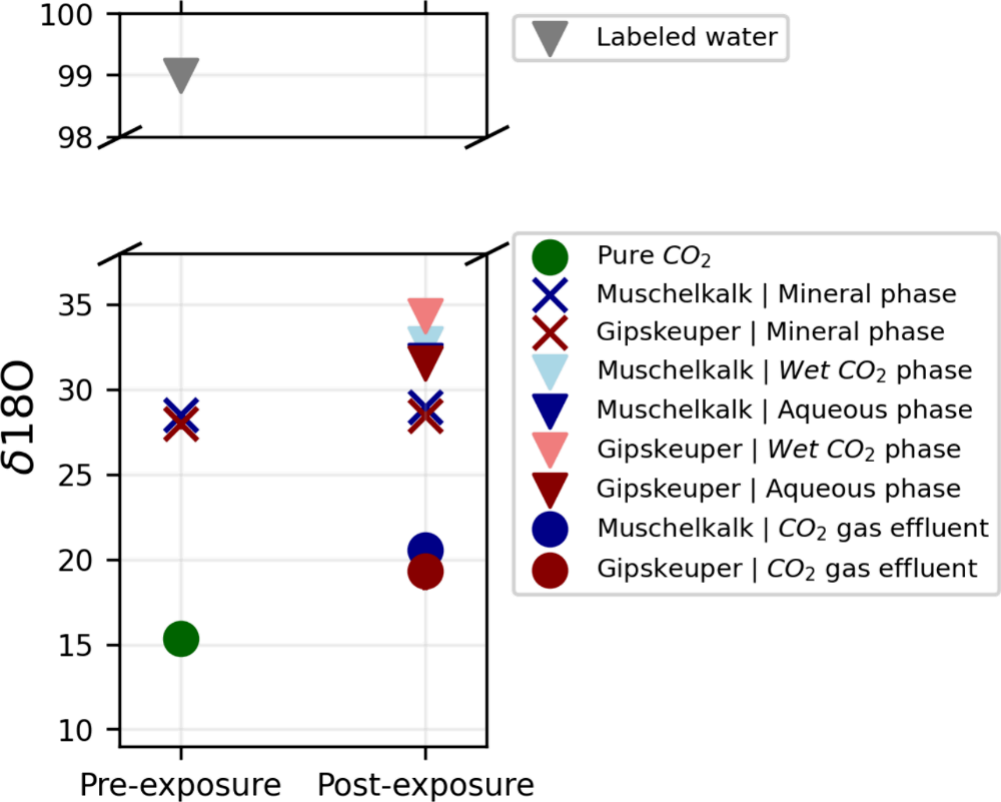
Pre- and postexperimental δ^18^O isotopic compositions of all samples. Each data point represents a measurement of six replicates.

Figure 7 shows the pre- and postexperiment δ^18^O compositions across all collected samples. Notably, the initial δ^18^O values were deliberately selected to cover a broad range from ∼15 for pure CO_2_ to 99 for the labeled water being used as a tracer. After the experiments, the δ^18^O values of all samples were modified and converged to a much narrower range of roughly 20 (for the CO_2_ gas effluents) to nearly 35 (for the condensed aqueous solutions). This observed postreaction convergence provides evidence of interactions and isotope fractionations among the three species (water, CO_2_, and rock) involved in the experiment.

**Figure 8:**
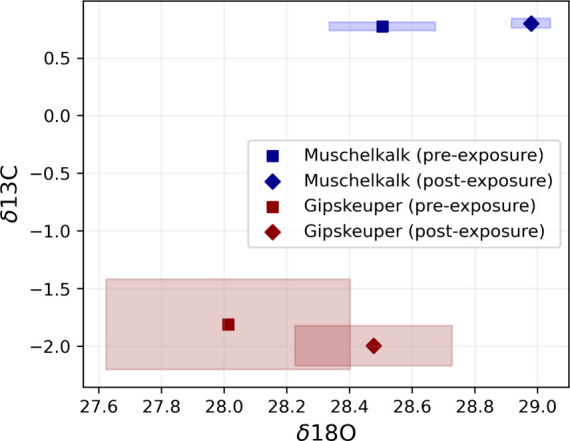
Isotopic composition (δ^18^O) versus δ^13^C) of the Muschelkalk and Gipskeuper samples before (squares) and after (diamonds) exposure to wet CO_2_. Each sample was measured using six replicates.

While the enrichment of ^18^O in the rock specimens after reactions is not obvious in Figure 7, it is much clearer in Figure 8, where the δ^18^O values are plotted against the δ^13^C values of the rock specimens. The data clearly show an enrichment of δ^18^O in the rock specimens following the reactions, a direct consequence of the addition of the δ^18^O-labeled water at the bottom of the bath reactors at the start of the experiments. The observed increase in δ^18^O aligns with the expected tracer behavior, providing clear evidence of oxygen isotope exchange during the reactions. In contrast, the δ^13^C values for both the Muschelkalk (reservoir) and Gipskeuper (caprock) samples remain consistent within the measurement uncertainty between the pre- and postreaction states. Additionally, the measurements reveal that the higher degree of mineralogical homogeneity in the Muschelkalk samples contributes to the improved reproducibility of isotopic analyses, as reflected by the tighter clustering of data points compared to the more heterogeneous Gipskeuper samples.

#### Mineral Alterations

Table 3 presents the mineral modal abundances (in wt %) of both rock specimens before and after the batch experiments. The Muschelkalk specimen starting material is composed of pure dolomite, whereas the Gispkeuper specimen also contains abundant anhydrite, with minor quantities of celestine and quartz. Comparison of the pre- and postexperiment XRD measurements confirms the anticipated absence of new mineral phases due to batch reactions involving wet CO_2_ in either the Gipskeuper or Muschelkalk sample. In addition, these data disclose a discernible amount of mineral-specific dissolution along the exposed surface of the Gipskeuper specimen, with dolomite abundances decreasing from 54.4% to 13.3% and anhydrite abundances increasing from 37.8% to 80.6%. In contrast, the XRD measurements of the Muschelkalk specimen indicate that it remains pure dolomite after the batch experiments, with no evidence of new mineral formation. Nonetheless, changes in grain size and other morphological features observed in the SEM micrographs suggest that dolomite in the Muschelkalk specimen underwent dissolution and reprecipitation. This observation is further supported by the ICP-AES results discussed in subsequent sections.

**Table 3:**
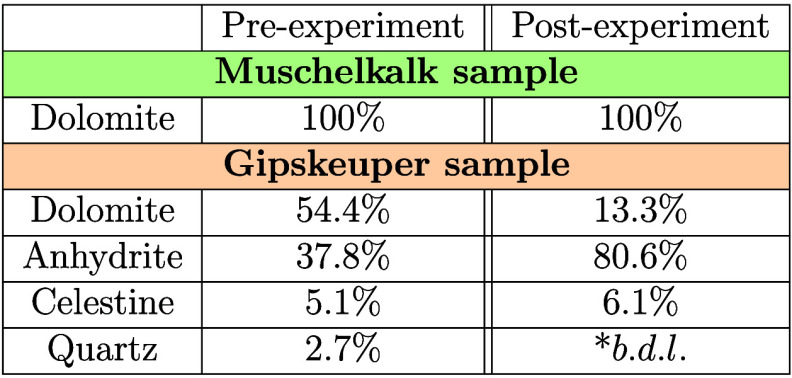
Crystallized Phases in Weight Percent, Derived from XRD Measurements^a^

a *b.d.l.* = below detection limit.

#### Ca/Mg ratio in Dolomite

Tables 4 and 5 document the molar compositions of both rock specimens, along with their weight percentage relative differences when compared to their pre-experiment states. These data illustrate that the 500 h batch reactions resulted in only minor alterations in the average chemical compositions of the exposed surfaces of both samples. The average weight percentage variability of all elements remains within −2.2% to 4.7% for the Muschelkalk sample and within −4.6% to 5.5% for the Gipskeuper specimen. Our SEM analyses suggest that dolomite was the main mineral to be dissolved, possibly saturating the fluid and thus impeding subsequent interactions with the less reactive mineral phases. Notably, the data indicate that trace elements not present in crystallized phases were predominantly dissolved during the experiments.

**Table 4:**
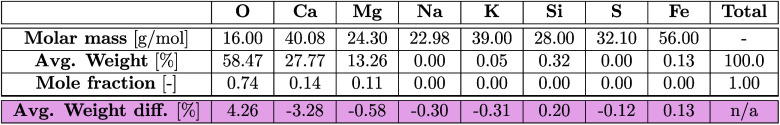
Element Composition of the Reacted Muschelkalk Specimen, Determined by SEM Scans, Accompanied by the Corresponding Average Weight Percentage Difference Relative to the Unreacted Composition

**Table 5:**
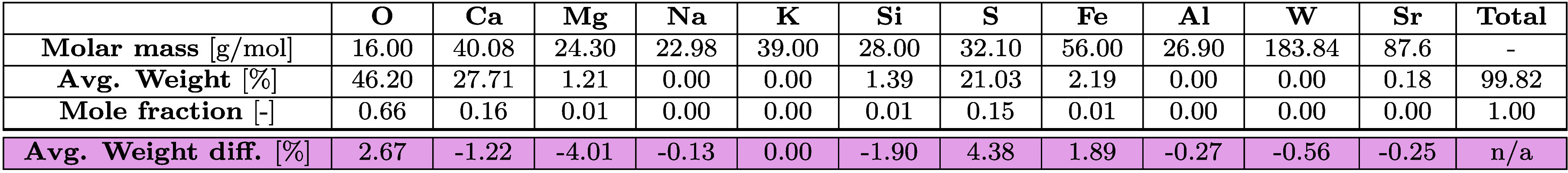
Element Composition of the Reacted Gipskeuper Specimen, Determined by SEM Scans, Accompanied by the Corresponding Average Weight Percentage Difference Relative to the Unreacted Composition

It is important to emphasize that our experiments were conducted in batch mode, wherein no forced advective flow was applied, as required for flow-through experiments. Therefore, mass transport during the experiments and up until effluent sampling is expected to be primarily driven by molecular diffusion, with the potential addition of buoyancy-driven convection if there is a density inversion in the fluid due to the presence of dissolved cations. Consequently, the overall reactivity of wet scCO_2_ could be significantly constrained by limited mass transport dynamics, approaching zero as the system reaches its chemical equilibrium. Flow-through experiments could exhibit heightened reactivity due to the continuous influx of a chemically unreacted and ion-depleted solution interacting with exposed mineral surfaces.^35,41^

The dissolution of dolomite followed by the precipitation of carbonate crystals with irregular morphology, as a direct consequence of exposure to wet scCO_2_, supports the observations made by Wang et al.^32^ However, an interesting yet unexplored aspect is revealed when examining the calcium-to-magnesium molar ratios in the solid phases of the reacted mineral surface. The Muschelkalk sample exhibits a slight decrease in the Ca/Mg ratio from originally 1.36 to 1.27 after the experiments. The subtle change in the Ca/Mg ratio underscores a limited reaction, a relative enrichment of magnesium, and a nonstoichiometric or incongruent dissolution of dolomite in our experiments. We attribute the limited reaction to the batch reaction mode. Although previous studies^91−93^ have revealed nonstoichiometric dolomite dissolution in aqueous reactions, we believe to be the first to confirm this phenomenon in the presence of wet scCO_2_. In contrast, the Gipskeuper sample displayed a more pronounced increase in its Ca/Mg ratio. Initially standing at 3.33, this ratio surged considerably to 10.95 upon the conclusion of the experiments. The disparate Ca/Mg ratios observed in the Gipskeuper sample stem from the preferential dissolution of the primary Ca–Mg-containing mineral (dolomite), whereas the exclusively Ca-rich mineral (anhydrite) demonstrates greater resistance to dissolution within wet scCO_2_ and thus remains in the solid phase. However, these results alone do not offer conclusive evidence, as the dissolution of minerals could have been induced by the wet scCO_2_ during the 500 h experiments and/or subsequently by the CO_2_-saturated water exsolved from the scCO_2_ during experiment termination. Nonetheless, we contend that the short experiment termination duration, compared to the 500 h experiment time frame, should not undermine our preceding interpretation on nonstoichiometric dissolution of dolomite in the presence of wet scCO_2_. To further substantiate our interpretation, the analysis of ion concentrations in fluid effluents, collected before and after the experiments, discussed in the subsequent section, is of paramount importance.

#### Incongruent Dissolution of Dolomite with Wet scCO_2_

**Figure 9:**
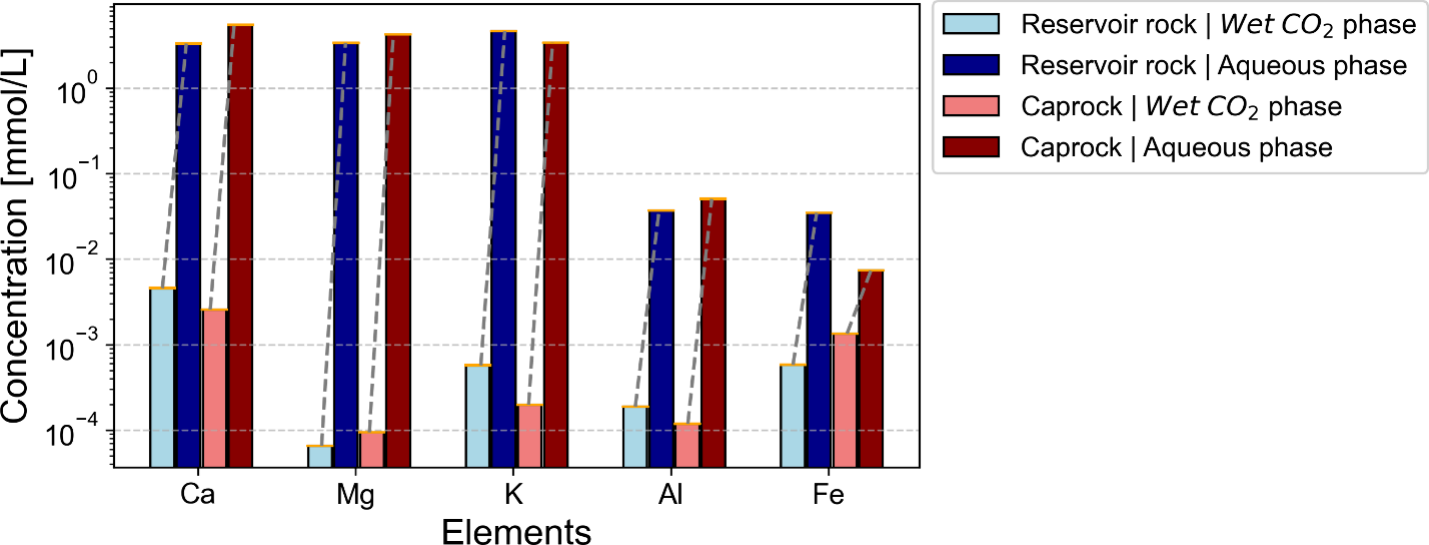
Dissolved cation concentrations in fluid effluents determined by inductively coupled plasma atomic emission spectroscopy (ICP-AES) measurements.

Aqueous solution samples were obtained from two distinct sources within the reactors: the condensed water sourced from the wet scCO_2_ effluent, while the batch reactions were taking place, and the residual water collected at the bottom of the reactors postreaction. These samples underwent analyses using inductively coupled plasma atomic emission spectroscopy (ICP-AES, Figure 9), and the resulting concentrations were corrected for the back-calculated original volumes within the reactor. The ion concentrations in samples from the wet scCO_2_ phase were initially more concentrated due to the release of gaseous CO_2_, which constituted most of the mixture volume. To accurately represent the original composition, these concentrations were corrected by considering the total volume of wet scCO_2_ that was sampled. A dilution factor of approximately 0.02 was calculated and applied uniformly to both batch reactors. In contrast, samples from the postreaction residual water underwent dilution, as not all the water present at the end of the experiments was initially dissolved in scCO_2_. For these samples, dilution factors of 3.4 and 4.12 were calculated and applied to the measurements obtained from the batch reactors containing the Muschelkalk and Gipskeuper specimens, respectively. By applying these correction factors, we ensure that the ion concentrations accurately reflect the original volumes and compositions of the samples in the reactors.

The residual aqueous phase at the reactor bottom exhibits significantly higher concentrations of major dissolved cations (i.e., Ca, Mg, and K), compared to the condensed water from the wet scCO_2_ effluents. The disparity in major cation concentrations typically ranges from 0.5 to 2 orders of magnitude, with concentrations being consistently higher in the residual water. The substantially elevated major cation concentration in the residual aqueous phase may be attributed to four potential sources: (1) settlement of fine particles detached from specimen surfaces during the placement of the specimen and scCO_2_ flashing during loading, (2) settlement of the condensed water film after coming into direct contact with the specimen’s surface, (3) settlement of condensed water droplets directly from the wet scCO_2_ phase without direct contact with the specimens, and (4) direct deposition of dissolved cations from the wet scCO_2_ phase. It is evident that without sources 1, 2, and 4, source 3 alone would yield lower cation concentrations in the residual water due to dilution. Source 4 is constrained by mass transport dynamics in batch-mode reactions. Therefore, we consider sources 1 and 2 as the most probable sources. Although it is currently challenging (if not impossible) to exactly identify each source’s contribution, the presence of dissolved cations in the sampled wet scCO_2_ effluents provides clear evidence of ion dissociation within the wet scCO_2_ phase and subsequent transport along the venting flow of this buoyant phase during the sampling process.

Analysis of the Ca/Mg molar ratios, obtained from fluid samples, further confirms the highly incongruent dissolution of dolomite in the wet scCO_2_ phase. Table 6 presents the Ca/Mg molar ratios initially found in the unreacted rock specimens as well as those obtained from the wet scCO_2_ phase and the aqueous phase at the conclusion of the experiments. After reactions, the condensed water samples from the wet scCO_2_ phase yield considerably higher Ca/Mg molar ratios, 41.48 and 31.03 for the Muschelkalk and Gipskeuper specimens, respectively. These ratios deviate significantly from their pre-experiment values (1.36 and 3.33 for the Muschelkalk and Gipskeuper specimens, respectively). This remarkable deviation confirms a substantial degree of incongruent reaction for dolomite dissolution with the wet scCO_2_, where calcium ions were preferentially leached by the wet scCO_2_ and magnesium remained in the solid phase, as shown in eqs 2a and 2b. In contrast, the residual water at the reactor bottom displays the lowest Ca/Mg molar ratio for both the Muschelkalk and Gipskeuper experiments. The ion concentration measurements of these aqueous samples resemble more closely the stoichiometric coefficients of the unreacted rock specimens.

**Table 6:**
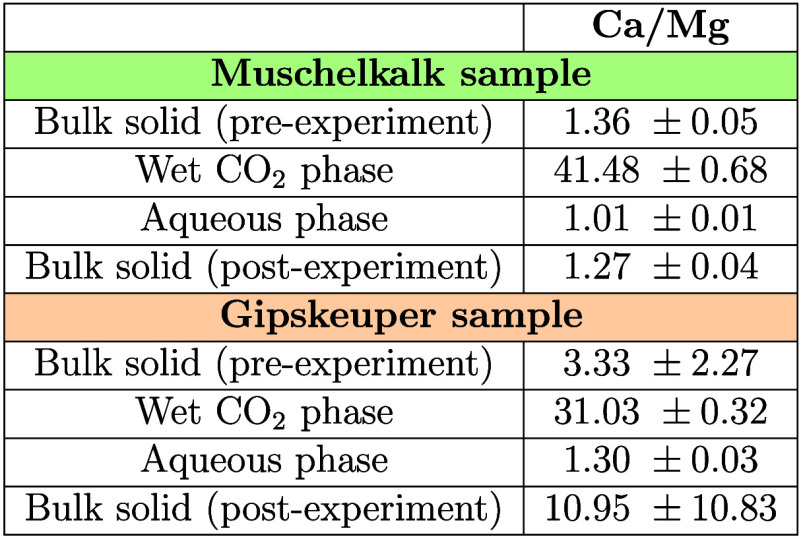
Stoichiometric Ratios of Calcium to Magnesium Moles in Solid and Fluid Samples along with Their Corresponding Standard Deviations

As indicated by previous studies that experimented with aqueous solutions and carbonate lithologies,^91−93^ incongruent dolomite dissolution in the Muschelkalk and the Gipskeuper samples is interpreted to have occurred in two stages as follows:
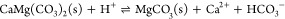
2a
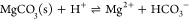
2b

**Figure 10:**
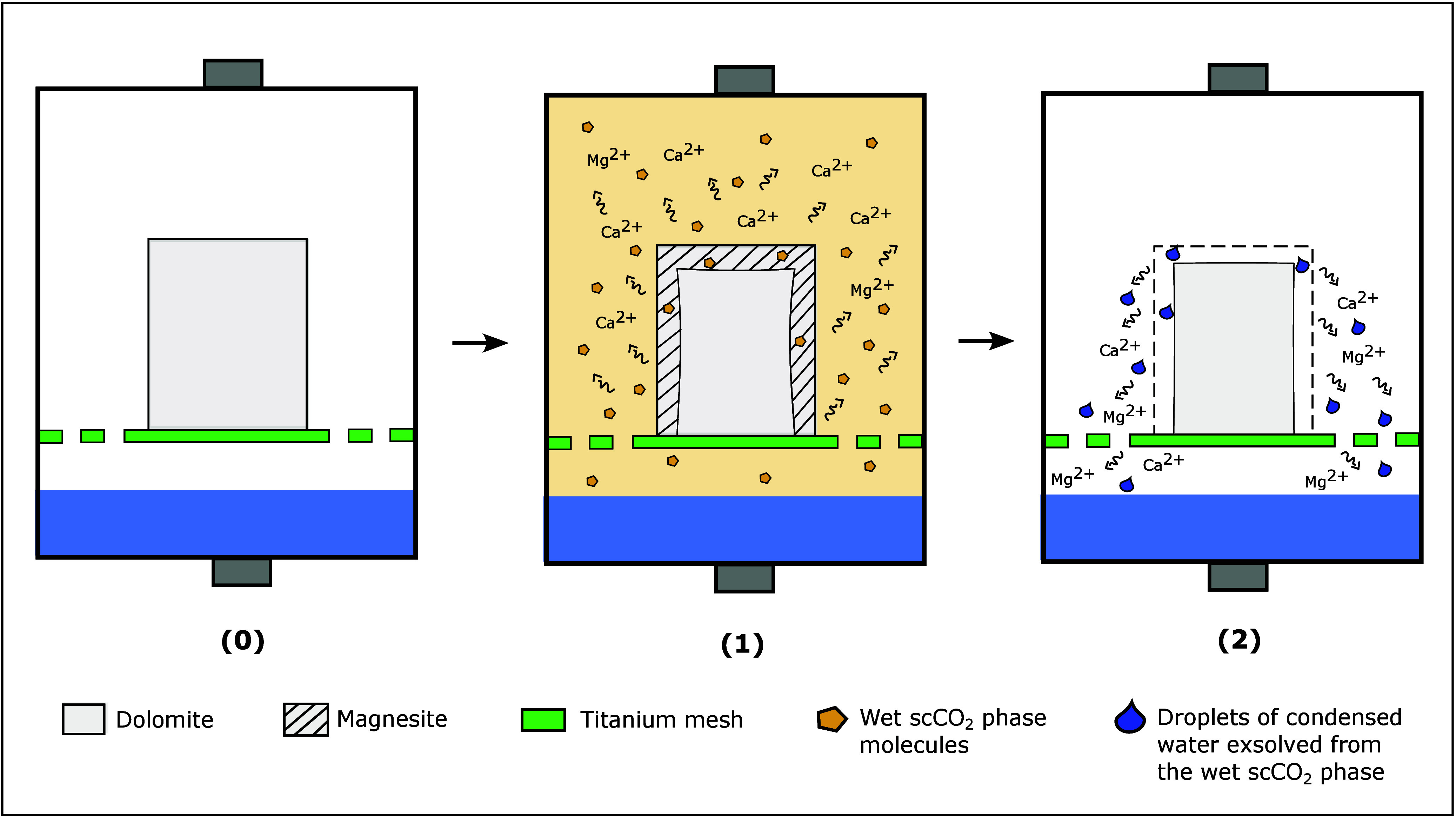
Interpretation of the two-stage dissolution process for a pure dolomite specimen. Stage 1: initial exposure to wet scCO_2_ under elevated pressure and temperature conditions promotes selective dissolution, resulting in a higher release of Ca^2+^ ions compared to that of Mg^2+^. Stage 2: upon experiment completion, condensed water exsolved from the wet scCO_2_ phase interacts with the dolomite, leading to congruent dissolution and a Ca/Mg ratio in the aqueous solution that resembles the bulk rock composition.

The considerably high Ca/Mg ratio observed in the wet scCO_2_ samples indicates that eq 2a predominantly occurred in the wet scCO_2_ phase, while eq 2b along with congruent dolomite dissolution was predominantly triggered by the exsolved aqueous solution (Figure 10). Previous studies by Rimmele et al.^80^ and Wang et al.^32^ documented carbonate dissolution induced by wet scCO_2_ using SEM analysis. However, their studies lacked simultaneous sampling of the nonaqueous phase, did not quantify cation molar ratios in the mineral and fluid phases, and were limited to an independent ex situ characterization of reacted products upon experiment termination. By contrast, our measurements and interpretations provide new insights into how wet scCO_2_ can drive the nonstoichiometric dissolution and transport of dolomite as a single-phase fluid. This new interpretation challenges the prevailing hypothesis that wet scCO_2_ can only be reactive through the partial exsolution of water, which forms a thin liquid film that accommodates mineral dissolution, solvates ionic species, and transports them in the vicinity of the mineral surface while remaining in situ.^33,75−78^ Additional support for our hypothesis can be found in the ion concentration measurements from the wet scCO_2_ flow-through experiments conducted by Choens et al.,^41^ which suggest direct ion migration into the wet scCO_2_. However, while Choens et al. attribute their observations to the “result of an aqueous phase reacting with the (rock) sample during exposure”, here we propose and provide evidence that mineral dissolution and transport can occur directly in the wet scCO_2_ phase as a nonaqueous reaction.

As depicted in Figure 10, the dissolution process involves two stages: an initial phase where wet scCO_2_ promotes selective dissolution of dolomite, releasing more Ca^2+^ than Mg^2+^ into the nonaqueous scCO_2_-rich phase, and a subsequent phase where condensed water interacts with dolomite, leading to congruent dissolution and a Ca/Mg ratio that mirrors the bulk rock composition. Further dissolution of dolomite in the scCO_2_-rich phase appears to have been self-limited by the rapid release of Ca^2+^ ions, potentially leaving magnesite (MgCO_3_) as a residual barrier, which could have inhibited further reactions with the wet scCO_2_ phase. However, the absence of MgCO_3_ in postexperimental XRD measurements combined with the significantly higher magnesium concentrations in the residual aqueous samples suggests that magnesite dissolved into the condensed aqueous solution during the final stages of the experiment. This interpretation highlights the dynamic and complex interplay between the nonaqueous and the aqueous phases in controlling mineral reactivity and dissolution mechanisms.

### Implications for CCS and CO_2_-Based Geothermal Energy

Our experimental results reveal a remarkably limited extent of mineral dissolution induced by water-bearing scCO_2_, even under elevated pressure and temperature conditions. Under the thermodynamic conditions studied, dolomite dissolution is characterized as incongruent, self-sealing, and self-inhibiting driven by the rapid release of Ca ions. This nonstoichiometric release appears to saturate the nonaqueous scCO_2_ phase, effectively halting further reactions with the remaining Mg-rich carbonates. These results suggest that secondary porosity generated through mineral dissolution in the presence of wet scCO_2_ may be self-sealing, potentially limiting progressive permeability changes in dolomitic reservoirs. Moreover, anhydrite dissolution in the wet scCO_2_ phase was found to be negligible, as corroborated by both XRD and SEM analyses. These observations highlight the geochemical robustness of dolomitic sedimentary reservoirs for sequestering and/or circulating water-bearing scCO_2_, particularly when they are capped by anhydrite-rich formations.

To contextualize the above in the broader framework of geothermal energy extraction, we numerically simulated dolomite dissolution in pure water, reaching chemical equilibrium under the same pressure and temperature conditions used in our experiments (i.e., 150 °C and 35 MPa), using the open-source chemical equilibrium solver *Reaktoro*.^94,95^ The simulations predicted equal solubility limits for calcium and magnesium at 0.07 mmol/L. Notably, these values are significantly higher than the corresponding Ca and Mg concentrations measured in the wet scCO_2_ samples from our experiments (see Figure 9). The *Reaktoro*-simulated solubility limits, for dolomite dissolution in pure water, are approximately 15 and 1000 times greater than our experimental concentrations for calcium and magnesium, respectively. These findings emphasize that utilizing scCO_2_ as a geothermal working fluid,^11,12,23^ even when saturated with water, would result in substantially fewer chemical reactions with dolomite compared to an equivalent geothermal system that employs water-based extraction.

To summarize, our findings indicate that although present and observed, the reactivity of wet scCO_2_ with carbonate rocks is minimal in laboratory-scale experiments and likely negligible in reservoir-scale CO_2_ injection operations. Consequently, the Muschelkalk and Gipskeuper formations in Switzerland appear to be well-suited for the long-term storage of wet scCO_2_ under elevated pressure and temperature conditions, as well as for geothermal energy extraction employing scCO_2_ as the energy transfer fluid. It is important to note that the potential impacts of advection-dominated transport and impurities dissolved in scCO_2_ (e.g., SO_2_, NO_*x*_, H_2_S, and NH_3_) are beyond the scope of this study. We recommend considering these factors in future research to reduce the risks and uncertainties of wet scCO_2_ injection under more realistic conditions.

## Conclusions

Derisking geological CO_2_ sequestration and CO_2_-based geothermal energy extraction requires a detailed understanding of reservoir and caprock reactivity to CO_2_–H_2_O mixtures in both aqueous and nonaqueous phases. Batch reaction experiments were conducted to investigate the reactivity of Muschelkalk and Gipskeuper rock formations in Switzerland under wet scCO_2_ exposure.

Our experimental results show that mineral dissolution took place in both the reservoir and caprock specimens to a limited but similar extent. Both rock specimens experienced a mass loss of less than 1% after roughly 500 h of fluid–mineral interactions. Euhedral dolomite crystals were dissolved, allowing for anhedral, smaller secondary carbonates to precipitate. Ion concentration analysis revealed that fluid–mineral interactions involving wet CO_2_-rich supercritical fluids are significantly less severe than those caused by CO_2_-bearing water under equivalent pressure and temperature conditions, despite the latter composition being in contact with the rocks for a considerably shorter time during our experiments.

Dolomite dissolution in wet scCO_2_ exhibited incongruent behavior, whereby calcium preferentially leached into the wet CO_2_, while magnesium tended to remain within the solid dolomite crystals. The substantial release of Ca^2+^ appears to have saturated the nonaqueous solution, inhibiting further dolomite dissolution. Upon termination of the experiments, the condensed aqueous solution appeared to dissolve dolomite congruently and to a greater extent, emerging as the primary driver for total mineral dissolution. Nonetheless, the detection of dissolved cations in the sampled wet scCO_2_ effluents constitutes, to the best of our knowledge, the first reported evidence of ion dissociation and transport caused by water continuously dissolved in the scCO_2_ phase as a single-phase fluid. Our findings highlight the need for further investigations into the dissolution dynamics and mechanisms governing wet scCO_2_ interactions with mineral assemblages.

## Supporting Information Available

Baseline information and petrophysical properties of the rock specimens, X-ray diffraction (XRD) patterns of rock specimens before and after fluid–mineral interactions, and simple mass balance calculation for determining the Ca/Mg molar ratio in dolomite using XRD and SEM data (PDF)
