# Supplementary material for: Reactivity of Wet scCO2 toward Reservoir and Caprock Formations under Elevated Pressure and Temperature Conditions: Implications for CCS and CO2-Based Geothermal Energy Extraction
Source: Energy Fuels. 2025 Jan 8;39(3):1679–93. doi: 10.1021/acs.energyfuels.4c04515 (PMC11770833; doi:10.1021/acs.energyfuels.4c04515)
Supplement: Supplementary file 1 — ef4c04515_si_001.pdf [file ef4c04515_si_001.pdf]

Supporting Information for Publication

Manuscript Title:
Reactivity of wet scCO_2 towards reservoir and caprock formations under elevated pressure and temperature conditions: Implications for CCS and CO_2-based geothermal energy extraction

Author List

Nicolás Rangel Jurado[1,6] (nicolas.rangel-jurado@ethz.ch),
Xiang-Zhao Kong[1] (xiangzhao.kong@eaps.ethz.ch),
Anna Kottsova[1] (anna.kottsova@eaps.ethz.ch),
Luiz Grafulha Morales[2] (luiz.morales@scopem.ethz.ch),
Ning Ma[3] (ning.ma@eaps.ethz.ch),
Federico Games[4] (federico.games@adterra.com),
Maren Brehme[1] (maren.brehme@eaps.ethz.ch),
Stefano M. Bernasconi[5] (stefano.bernasconi@eaps.ethz.ch),
Martin O. Saar[1] (martin.saar@eaps.ethz.ch)

Affiliations

[1]Geothermal Energy and Geofluids Group, Institute of Geophysics, Department of Earth and Planetary Sciences, ETH Zurich, Zurich, 8092, Switzerland
[2]Scientific Centre for Optical and Electron Microscopy (ScopeM), ETH Zurich, Zurich, 8093, Switzerland
[3]Institute of Geochemistry and Petrology, Department of Earth and Planetary Sciences, ETH Zurich, Zurich, 8092, Switzerland
[4]Ad Terra Consultancy, Geneva, 1208, Switzerland
[5]Climate Geology, Geological Institute, Department of Earth and Planetary Sciences, ETH Zurich, Zurich, 8092, Switzerland
[6]Computational Geoscience, Geothermics, and Reservoir Geophysics, RWTH Aachen, Aachen, 52074, Germany

Corresponding Author: N. Rangel Jurado[1,6] and X-Z. Kong[1]

Baseline information and petrophysical properties of the rock specimens

Table 1: Petrophysical properties of rock specimens prior to interaction with wet $scCO_2$ under elevated pressure and temperature conditions.

**Label**	**728.18**	**822.72A**
**Wellbore**	Benken	Weiach
**Lithological unit**	Gipskeuper (Caprock)	Muschelkalk (Reservoir)
**Density** [g/cm^3]	2.95	2.18
**Mass** [g]	40.18	29.41
**Porosity** [%]	0.6	22
**Permeability** [m^2]	2.3×10^{-20}	1.64×10^{-15}

X-ray diffraction patterns of rock specimens before and after fluid-mineral interactions

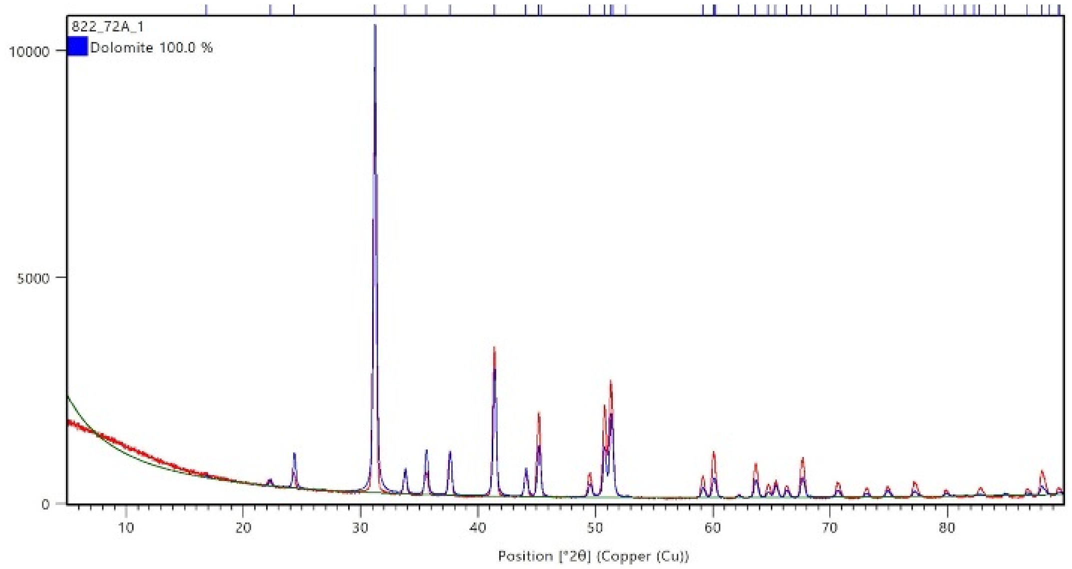

Figure 1: XRD spectra for specimen 822.72A (Muschelkalk) before experimental exposure.

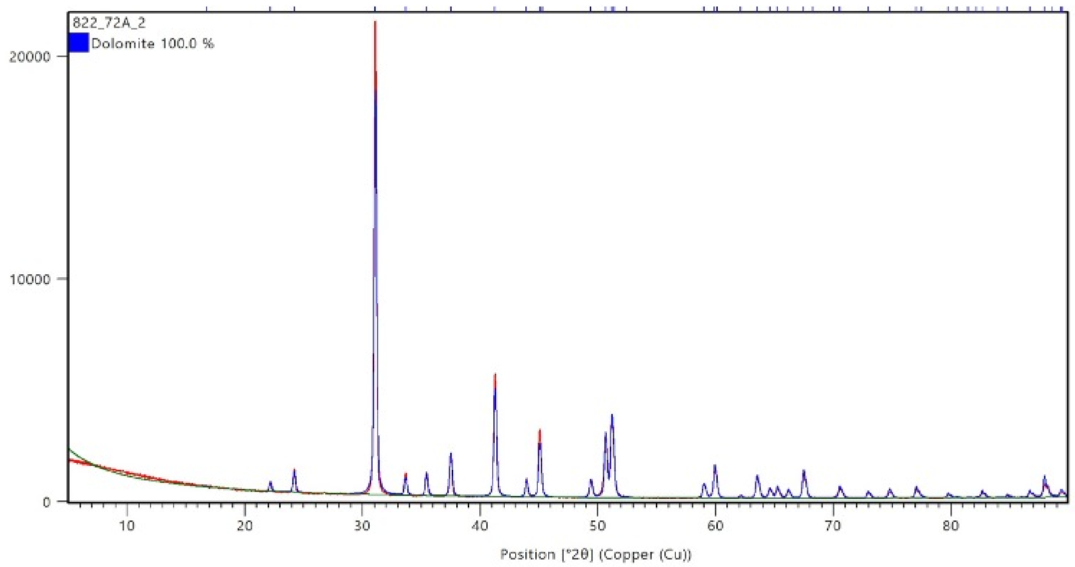

Figure 2: XRD spectra for specimen 822.72A (Muschelkalk) after experimental exposure.

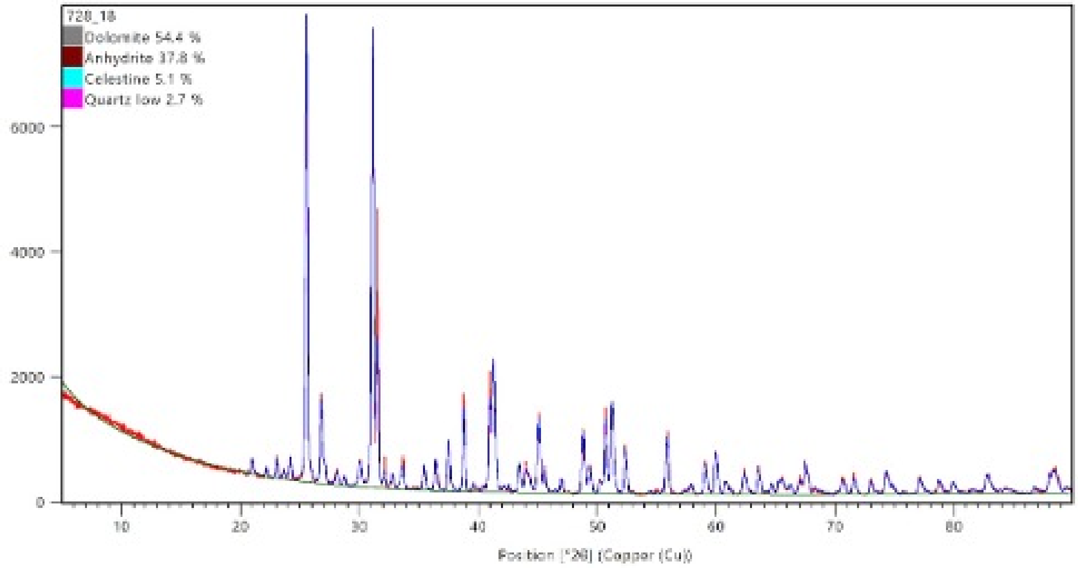

Figure 3: XRD spectra for specimen 728.18 (Gipskeuper) before experimental exposure.

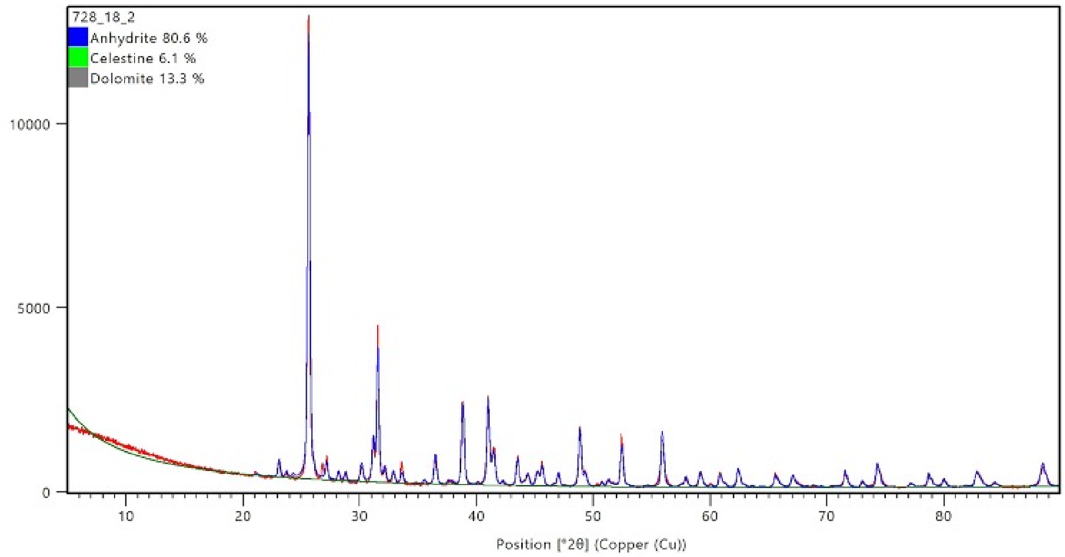

Figure 4: XRD spectra for specimen 728.18 (Gipskeuper) after experimental exposure.

Mass balance calculation for determining the Ca/Mg molar ratio in dolomite using XRD and SEM data

This appendix presents a simple mass balance calculation for determining the Ca/Mg molar ratio in the dolomite phase of the Gipskeuper specimen.

In our calculation, the dolomite formula is assumed to be $\mathrm{Ca}_x\mathrm{Mg}_y(\mathrm{CO}_3)_2$, where the stoichiometric coefficients x and y need to be determined, however, $x+y=2$ due to charge neutrality. The molar mass of dolomite is thus expressed as $\mathrm{M}_{\mathrm{dolomite}} = 40x + 24.3y + 120$ [g/mol]. The chemical formula of anhydrite is CaSO_4 with a molar mass of $\mathrm{M}_{\mathrm{anhydrite}} = 136.14$ [g/mol].

For one unit weight (e.g. 1 g) of Gipskeuper specimen, the molar amount of calcium (n_{Ca}) can be calculated using the weight percentages of dolomite, f_{dolomite}, and anhydrite, $f_{\mathrm{anhydrite}}$, obtained from XRD measurements (see Table 4 in main text):

$$n_{\mathrm{Ca}} = x\frac{f_{\mathrm{dolomite}}}{M_{\mathrm{dolomite}}} + \frac{f_{\mathrm{anhydrite}}}{M_{\mathrm{anhydrite}}},$$

Similarly, the molar amount of magnesium (n_{Mg}) is calculated as:

$$n_{\mathrm{Mg}} = y\frac{f_{\mathrm{dolomite}}}{M_{\mathrm{dolomite}}}.$$

Together with the SEM analysis of Ca/Mg element ratio in the bulk Gipskeuper specimen (see tab:ICP-AES$_{C}a/Mg_{r}atios), thestoichiometriccoefficientsx$ and y are then determined.

The pre-experimental measurements, where $f_{\mathrm{dolomite}} = 54.4\%$, $f_{\mathrm{anhydrite}} = 37.8\%$, and $n_{\mathrm{Ca}}/n_{\mathrm{Mg}} = 3.33$, yield $x = 2.66y - 0.51$. Substituting it into the charge neutrality in the dolomite phase ($x + y = 2$), we find:

$$x = 1.31 \quad \text{and} \quad y = 0.69,$$

yielding a Ca/Mg ratio of $x/y = 1.90$ in dolomite before the reactions.

The post-experimental measurements, where $f_{\mathrm{dolomite}} = 13.3\%$, $f_{\mathrm{anhydrite}} = 80.6\%$, and $n_{\mathrm{Ca}}/n_{\mathrm{Mg}} = 10.95$, yield $x = 3.55y - 1.92$. Substituting it into the charge neutrality in the dolomite phase ($x + y = 2$), we obtain:

$$x = 1.14 \quad \text{and} \quad y = 0.86,$$

yielding a Ca/Mg ratio of $x/y = 1.32$ in dolomite after the reactions.

Our simple calculations support the observation of a Ca/Mg molar ratio reduction in the dolomite phase, corroborating the non-stoichiometric dissolution of dolomite as indicated by ion concentration measurements.
